# Whole-Genome Resequencing Points to Candidate DNA Loci Affecting Body Temperature under Cold Stress in Siberian Cattle Populations

**DOI:** 10.3390/life11090959

**Published:** 2021-09-13

**Authors:** Alexander Igoshin, Nikolay Yudin, Ruslan Aitnazarov, Andrey A. Yurchenko, Denis M. Larkin

**Affiliations:** 1 Institute of Cytology and Genetics SB RAS, 630090 Novosibirsk, Russia; igoshin@bionet.nsc.ru (A.I.); yudin@bionet.nsc.ru (N.Y.); autrus@bionet.nsc.ru (R.A.); jurchenko@bionet.nsc.ru (A.A.Y.); 2 Department of Natural Sciences, Novosibirsk State University, 630090 Novosibirsk, Russia; 3 Department of Comparative Biomedical Sciences, Royal Veterinary College, University of London, London NW1 0TU, UK

**Keywords:** body temperature maintenance, cold adaptation, cattle, whole-genome resequencing

## Abstract

Despite the economic importance of creating cold resilient cattle breeds, our knowledge of the genetic basis of adaptation to cold environments in cattle is still scarce compared to information on other economically important traits. Herein, using whole-genome resequencing of animals showing contrasting phenotypes on temperature maintenance under acute cold stress combined with the existing SNP (single nucleotide polymorphism) functional annotations, we report chromosomal regions and candidate SNPs controlling body temperature in the Siberian cattle populations. The SNP ranking procedure based on regional *F*_ST_ calculations, functional annotations, and the allele frequency difference between cold-tolerant and cold-sensitive groups of animals pointed to multiple candidate genes. Among these, *GRIA4*, *COX17*, *MAATS1*, *UPK1B*, *IFNGR1*, *DDX23*, *PPT1*, *THBS1*, *CCL5*, *ATF1*, *PLA1A*, *PRKAG1,* and *NR1I2* were previously related to thermal adaptations in cattle. Other genes, for example *KMT2D* and *SNRPA1,* are known to be related to thermogenesis in mice and cold adaptation in common carp, respectively. This work could be useful for cattle breeding strategies in countries with harsh climates, including the Russian Federation.

## 1. Introduction

Extreme environmental temperatures are a growing challenge for animal agriculture in light of climate changes and globalization [1,2,3,4]. While there are cold-adapted cattle [5,6], goat [7], pig [8], horse [9], chicken [10,11] and other livestock and poultry breeds, cold tolerance in most livestock has not been actively selected for. With the development of genetic tools, it is now possible to genetically modify animals to introduce traits of interest including cold resistance, if the exact genetic basis is known. Given that they are one of the world’s major meat and milk sources [12], with some populations adapted to cold environments, cattle could represent a promising livestock for such endeavors.

The genetics of cold adaptation in cattle have been recently studied both at the genome [6,13,14,15] and transcriptome [16,17,18] levels. Using whole-genome genotyping data, we revealed *GRIA4* as a promising candidate associated with body temperature maintenance under extremely low temperatures in Siberian cattle populations [19]. The same gene has been recently reported as a candidate for heat stress resilience in Australian Holsteins [20], implying its possible contribution to extreme temperature adaptations in general. Despite the abovementioned efforts, the current knowledge of the genetics behind cold resistance is scarce compared to many other economically important traits [21].

To find candidate functional variants contributing to cold adaptation in cattle, in this work we utilized a comprehensive set of cattle SNPs of a known functional type (e.g., splicing QTLs, expression QTLs, conserved sites and so on) scored based on their ability to explain variation in 34 complex traits [22]. This Functional-And-Evolutionary Trait Heritability (FAETH) score could help to identify the most influential variants contributing to cold adaptation as this phenotype is influenced by multiple physiological and morphological features. We performed a search for such SNPs and looked at their allele frequency differences in a set of animals expressing contrasting temperature maintenance phenotypes, with samples being obtained from our previous experiment [19].

## 2. Materials and Methods

The phenotype measurement procedure was described in our previous study [19]. Briefly, temperature sensors attached to RFID ear tags were placed into the ear canals of nearly two hundred animals for two weeks in winter and transmitted measurements to a personal computer. After all the measurements were completed, the area under the curve of the in-ear temperature over the interval of the five coldest (down to −32 °C) days was calculated for each animal and further used as a proxy for the body temperature maintenance phenotype. DNA extracted from blood using cell lysation and phenol-chloroform extraction [19] from twelve animals of Hereford (7) and Kazakh Whiteheaded (5) breeds expressing extreme values for contrasting phenotypes (six individuals per group) and balanced for breed representation in each group was used in this study (Table S1). Samples were sequenced using Illumina Hiseq4000 technology at Novogene Co., Ltd. (Hong Kong, China) (150 bp paired reads, library insert size 350 bp) to ~50 Gbp each. Cleaned reads were mapped to the reference cattle genome assembly (Btau6) using BWA-MEM (Wellcome Trust Sanger Institute, Cambridge, UK) [23] with default parameters resulting in 99.7% of reads being mapped. Alignment post-processing and variant calling were done following the Genome Analysis Toolkit (GATK v. 3.8, Broad Institute, Cambridge, MA, USA [24]) pipeline. For each raw BAM file, we marked duplicate reads with Picard (v. 1.69) using the tool MarkDuplicates (http://broadinstitute.github.io/picard/, accessed date: 12 September 2021). Next, we performed a base quality score recalibration (using cattle known variants: dbSNP148). We followed the best practice guidelines [24] recommended for variant discovery and genotyping using GATK v.3.8 with default parameters. First, genotype likelihoods were calculated separately for each sequenced animal using HaplotypeCaller (Broad Institute, Cambridge, MA, USA), which resulted in files in the gVCF (genomic Variant Call Format) format for each sample. Subsequently, GenotypeGVCFs (Broad Institute, Cambridge, MA, USA) was applied to genotype polymorphic sequence variants for 12 samples simultaneously. The average filtered sequence coverage obtained was 10X. Filtering of SNPs for quality (“hard filtering”) has been applied using the following parameters: (i) variant confidence/quality by depth  <  2; (ii) RMS mapping quality (MQ)  <  40.0; (iii) Phred-scaled *p*-value using Fisher’s exact test to detect strand bias  >  60; (iv) Z-score from the Wilcoxon rank sum test of alternative vs. reference read MQs (MQRankSum)  <  −12.5; and (v) Z-score from the Wilcoxon rank sum test of alternative vs. reference read position bias (ReadPosRankSum)  <  −8. The thresholds for these parameters were adopted from GATK Best Practices [24]. INDEL variants were removed, resulting in a VCF file containing 17,561,905 high-quality SNPs. The transition/transversion rate for this set of SNPs was 2.04. After additional filtering steps (Figure S1), these data were used for *F*_ST_ calculations between groups of animals.

Calculations of *F*_ST_ [25] between the cold-‘sensitive’ and -‘tolerant’ groups were conducted with VCFtools v.0.1.13 (Wellcome Trust Sanger Institute, Cambridge, UK) [26] both for single SNPs (“--weir-fst-pop” option) and for sliding windows (“--fst-window-size 50,000 --fst-window-step 25,000”).

To identify candidate SNPs segregating together with cold-sensitivity phenotypes, we combined the data on the window-based weighted *F*_ST_, FAETH score of individual SNPs and individual SNP allele frequency differences between the two groups of animals. Each SNP with this information was annotated with three fractional (i.e., ranging from 0 to 1) ranks so that the higher the value was, the closer to zero the rank of the SNP would be. The *F*_ST_ rank for each SNP was calculated based on the rank of its higher-valued (as nearly all the variants are harbored by two overlapping intervals) window amongst 100,443 regions resulting from the window-based analysis. The ranks for allele frequency differences were calculated based on the list of 16,647,833 autosomal SNPs having at least three successfully called genotypes in each of the two contrasting groups. FAETH score ranks were calculated based on the abovementioned SNPs having FAETH annotation (8,836,652 SNPs), of which 0.35% were novel according to cattle dbSNP148. The ranks were summed up, and SNPs were sorted based on the resulting sum (Table S4). Top ranked (sum of ranks < 0.1) polymorphisms were annotated using the NGS-SNP annotation system [27] and Variant Annotation Integrator [28]. The annotation of variants with their corresponding genes was performed based on Bos_taurus.UMD3.1.94.gtf (Ensembl) and bosTau8.refGene.gtf (UCSC) files. Searching for candidate genes was carried out by manually investigating available information in Google and PubMed. The main key phrases and keywords were “body temperature”, “cold temperature”, “cold adaptation”, “thermoregulation”, “thermogenesis” and “hair follicle”. These key phrases/words were coupled with different target species names (“cattle”, “sheep”, “goats”, “pigs”, “horses”) or used alone.

## 3. Results

A single-point SNP *F*_ST_ analysis resulted in 11,908 biallelic SNPs, of which 63 (0.53%) were novel, from 829 genes with *F*_ST_ values ≥ 0.7. Three hundred ninety-six loci from 53 genes had *F*_ST_ = 1 (Table S2). A windows-based *F*_ST_ of 100,443 overlapping 50 Kbp autosomal regions resulted in the highest weighted *F*_ST_ value of 0.54 (Table S3) in the region BTA12:38850001-38900000 containing no known genes. Out of 829 genes from the single-point analysis, 92 were found among 212 genes from the top 0.5% windows-based results.

**Figure 1:**
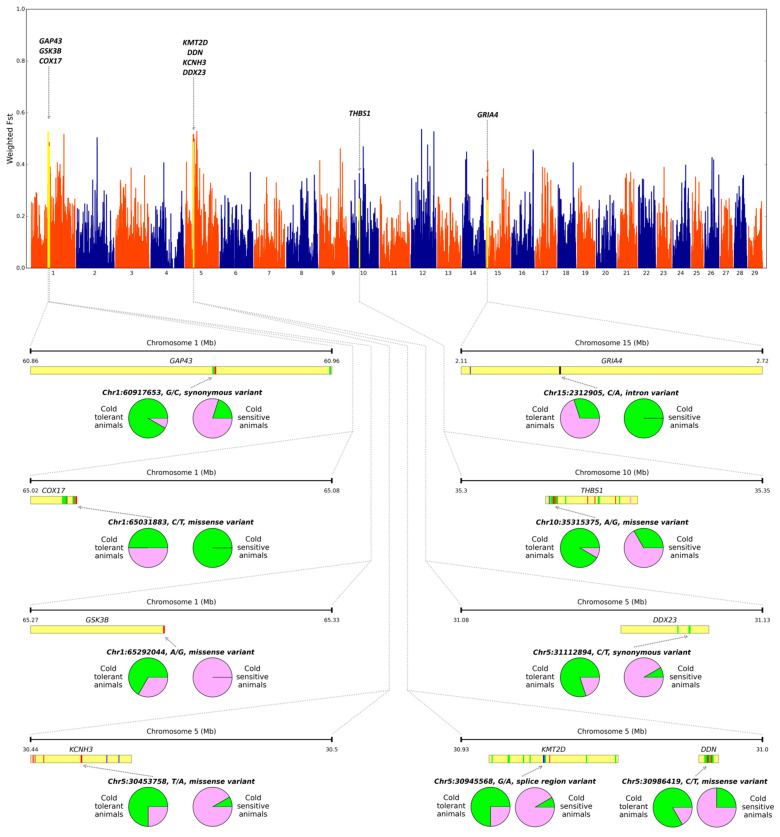
Examples of candidate variants for cattle body temperature maintenance during cold stress and their genomic location. Pie charts depict allele frequencies in contrasting groups. The green color shows the reference allele. Red, green, blue and pink barcodes stand for missense, synonymous, splice region and 3′-UTR variants, respectively.

The SNP ranking procedure (Table S4) resulted in 17,391 SNPs (six novel (0.03%)) with a sum of ranks < 0.10, of which 635 were synonymous variants, 258 were missense variants and 91 were splice region variants. After a manual literature search, 30 candidate genes were found. For each gene, at least one candidate variant was proposed (Table S5; some examples in Figure 1). For 13 genes there is evidence of contribution to temperature adaptations in bovine species (Table 1).

## 4. Discussion

Among the top results of the SNP ranking we found multiple genes previously related to thermal adaptations in cattle, close *Bovinae* and other species (Table 1). Among them, *DDX23* harboring a synonymous variant with the lowest sum of ranks shows an almost 2.5-fold transcriptional upregulation in tropically adapted Sahiwal cattle in comparison to high-altitude adapted Ladakhi cattle [29]. Additionally, *DDX23* is upregulated under cold stress in common carp (*Cyprinus carpio* L.) and belongs to core cold response genes in this species [30]. Although synonymous codon substitutions do not lead to amino acid change, they could affect the cellular level of coded protein or, in some cases, its structure [45] or be linked to genetic variants of other types. Another candidate is *THBS1,* with two high-rank missense variants. This gene is involved in cattle adaptation to both cold [18] and hot [33] climates as well as involved in cold adaptation in pigs [34]. According to a recent study on mice, *THBS1* contributes to a molecular pathway regulating the browning of adipocytes [46], which is essential for adaptive thermogenesis [47]. It is worth noting that among the top ranked SNPs we found rs207668622 from *GRIA4*, a gene identified as a major candidate in our previous study of a wider Siberian cattle population using genotyping arrays [19]. This variant falls into the intron four of *GRIA4*.

For some genes, we found additional evidence in species phylogenetically close to cattle. For example, according to Swain and colleagues, variations in *TMBIM6* affect the rectal temperature in Indian goats [48], while *FKBP11* is a candidate for involvement in seasonal adaptations in reindeer (*Rangifer tarandus*) [49]. There are several genes that are related to thermoregulation or cold adaptation in non-ungulate species. For example, *SLC10A2* is involved in bile acid metabolism under cold stress in mice [50]. According to Pereira–da–Silva and colleagues [51], *GAP43* is upregulated in the hypothalamus of rats exposed to cold. *KMT2D* contributes to the thermogenic adipose program in mice [52]. *SNRPA1* is involved in adaptation to cold in common carp (*Cyprinus carpio*) [53].

Interestingly, many genes from our candidate set are also associated with various economically important traits in cattle. For example, according to CattleQTLdb [21], *MAATS1* is related to reproduction (inseminations per conception and sperm concentration). *GRIA4* is associated with reproductive (first service conception and inseminations per conception) as well as milk traits (milk fat percentage and milk fat yield). A likely explanation for this is twofold: genes involved in cold adaptation contribute to various metabolic processes [54,55] and thus have pleiotropic effects, or there is a trade-off between cold adaptation and other traits requiring an extensive energy expenditure. It is also worth noting that the number of association studies in cattle which involve thermoregulation or cold adaptation phenotypes is limited, meaning that relevant gene functions could still be unknown.

## 5. Conclusions

This study, which utilizes the whole-genome resequencing of animals to show contrasting phenotypes on temperature maintenance under acute cold stress and functional genome annotations, reveals multiple candidate loci controlling the body temperature in the Siberian cattle population. The top genome intervals based on the weighted *F*_ST_ analysis contained many genes with a thermoregulatory and/or cold adaptation function. This work would be useful for cattle breeding in countries with a cold climate as it points to genetic variants segregating in cold-adapted populations, to be tested in breeding and genomic selection programs.

## Figures and Tables

**Table 1:** Genes with a known role in thermal adaptations in cattle and other species and their candidate nucleotide variants.

Gene	SNP Position (UMD3.1), (RefSNP)	Reference/Alternate Allele	Sum of Ranks	Reference Allele Frequency		Functional Class	Literature Evidence
				Cold-Sensitive Group	Cold-Tolerant Group		
*DDX23*	Chr5:31,112,894 (rs108955444)	C/T	0.002	0.08	0.80	synonymous variant	Climate adaptation in cattle [29], cold adaptation in common carp [30]
*MAATS1*	Chr1:65,062,344 (rs43234266)	T/C	0.01	1.00	0.13	synonymous variant	Adaptation of cattle to tropical climates [31]
*GRIA4*	Chr15:2,312,905 (rs207668622)	C/A	0.01	1.00	0.30	intron variant	Cold [19] and heat [20] adaptations in cattle
*COX17*	Chr1:65,031,883 (rs208045948)	C/T	0.02	1.00	0.50	missense variant	Adaptation of cattle to tropical climates [31], cold adaptation in Antarctic icefish [32]
*THBS1*	Chr10:35,315,375 (rs43707861)	A/G	0.02	0.33	0.92	missense variant	Cold [18] and heat [33] adaptations in cattle, cold adaptation in pigs [34]
	Chr10:35,320,988 (rs17870352)	A/G	0.02	0.33	0.90	missense variant	
*CCL5*	Chr19:14,825,116 (rs208398974)	C/T	0.02	0.25	1.00	synonymous variant	Cold adaptation in cattle [18], thermoregulation in rats [35]
*UPK1B*	Chr1:64,592,185 (rs43652277)	A/G	0.02	0.10	0.63	missense variant	Adaptation of cattle to tropical climates [31]
*PLA1A*	Chr1:64,966,636 (rs43233262)	C/A	0.03	0.00	0.83	intron variant	Adaptation of buffaloes to heat stress [36]
*NR1I2*	Chr1:65,236,459 (rs43235975)	T/C	0.04	0.00	0.42	synonymous variant	Adaptation of cattle to heat stress [31,37], cold stress response in mice [38]
*ATF1*	Chr5:29,271,337 (rs210280224)	A/G	0.06	0.00	0.63	downstream gene variant	Adaptation of cattle to heat stress [39], regulation of brown adipose tissue thermogenesis in mammals [40]
*PRKAG1*	Chr5:30,981,551 (rs29002398)	T/C	0.06	0.08	0.83	3′-UTR variant	Adaptation of cattle to heat stress [41], regulation of brown adipose tissue thermogenesis in mammals [42]
*IFNGR1*	Chr9:76,093,074 (rs41569368)	T/G	0.06	0.83	0.33	synonymous variant	Cold adaptation in cattle [18]
*PPT1*	Chr3:106,629,521 (rs42791314)	T/C	0.07	0.30	0.88	missense variant	Heat adaptation in cattle [43], thermoregulation in mice [44]

## Data Availability

The raw sequencing data for 12 animals are available from NCBI SRA under the BioProject accession number PRJNA762180.

## Supplementary Materials

The following are available online at https://www.mdpi.com/article/10.3390/life11090959/s1, Table S1: Information on animal phenotypes and breeds; Table S2: Top SNPs by single locus *F*_ST_ values; Table S3: The results of region-based *F*_ST_ analysis; Table S4: Top SNPs by sum of fractional ranks and their functional annotation; Table S5: Candidate variants and genes; Figure S1: The schematic representation of the SNP detection and analysis steps.
