# Supplementary figures and images for: Whole-Genome Resequencing Points to Candidate DNA Loci Affecting Body Temperature under Cold Stress in Siberian Cattle Populations
Source: Life (Basel). 2021 Sep 13;11(9):959. doi: 10.3390/life11090959 (PMC8467296; doi:10.3390/life11090959)

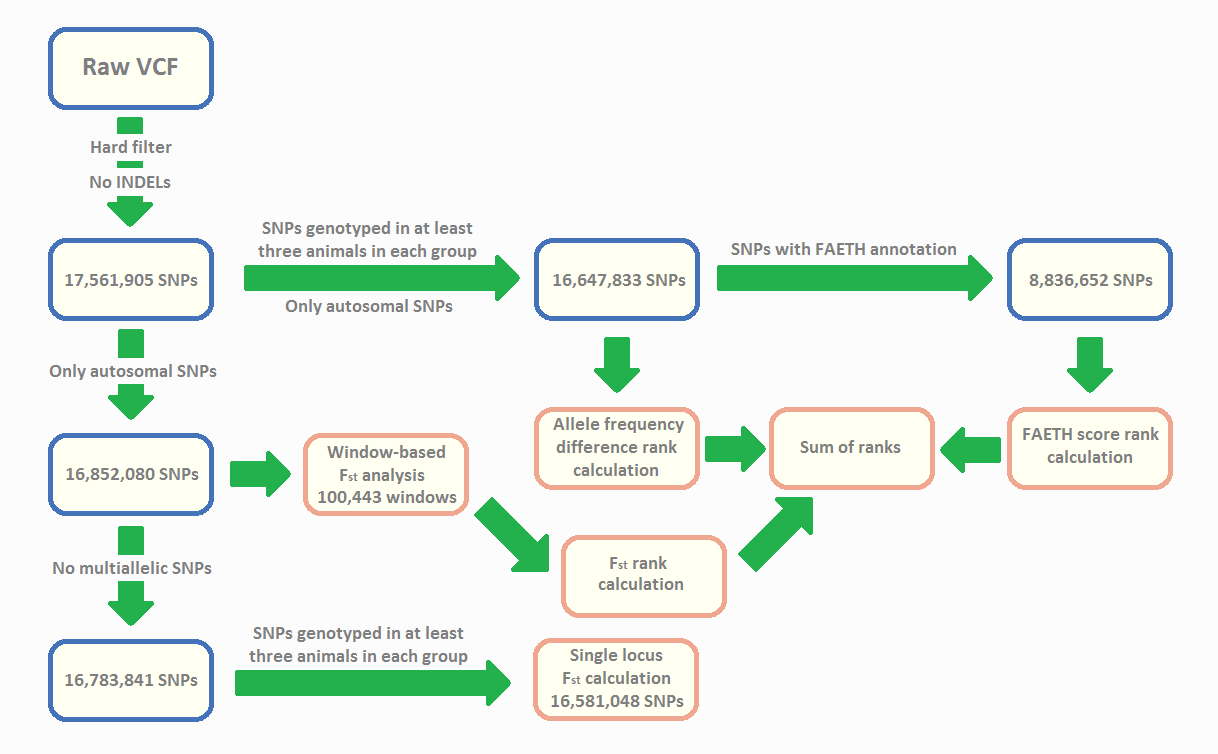

Supplement: Supplementary file 1 [file life-11-00959-s001.zip › Figure S1.tif]
